# Commentary: GLYX-13 Ameliorates Schizophrenia-Like Phenotype Induced by MK-801 in Mice: Role of Hippocampal NR2B and DISC1

**DOI:** 10.3389/fnmol.2018.00315

**Published:** 2018-09-05

**Authors:** Yuanyi Xie, Xu-Feng Huang

**Affiliations:** Illawarra Health and Medical Research Institute and School of Medicine, University of Wollongong, Wollongong, NSW, Australia

**Keywords:** GluN2B, DISC1, dopamine D2 receptor, schizophrenia, NMDAR agonist

It is with great interest we have read the article “GLYX-13 Ameliorates Schizophrenia-Like Phenotype Induced by MK-801 in Mice: Role of Hippocampal NR2B and DISC1” (Zhou et al., 2018).

Interestingly, this study showed that GLYX-13 prevents hippocampal N-methyl-D-aspartate receptor subtype 2B—Disrupted in schizophrenia 1 (GluN2B-DISC1) signaling and behavioral changes induced by schizophrenia-mimetic drug MK801 in mice. To confirm GluN2B directly regulating DISC1, these researchers showed that the effects of GLYX-13 were vanished after GluN2B knockdown in mice.

GLYX-13 is an amidated tetrapeptide. Studies have shown that GLYX-13 can specifically bind to the glycine site of N-methyl-D-aspartate receptors (NMDARs) and act as a partial agonist of GluN2B-containing NMDARs (Moskal et al., 2005; Stanton et al., 2009). GLYX-13 improves learning and increases the magnitude of LTP in rat hippocampus (Zhang et al., 2008). Both GLYX-13 and ketamine have antidepressant-like effects, while GLYX-13 has fewer psychotomimetic side effects than ketamine in both humans and rats (Burgdorf et al., 2013; Moskal et al., 2014). According to previous study, both GLYX-13 and ketamine are able to increase cell surface protein expression of GluN2B in rats (Burgdorf et al., 2013). This might help to explain similar antidepressant effects of these two compounds. However, the mechanism how GLYX-13 causes less side effects is still unclear. Interestingly, memantine, another weak NMDAR modulator, also shows fewer side effects than other NMDAR antagonists in treating Alzheimer's disease (Parsons et al., 1999). Therefore, it is important for the treatment of psychiatric disorders to understand how GLYX-13 and other NMDAR modulators affect the neuronal system in psychiatric disorders without causing serious side effects.

Although GluN2B-containing NMDAR hypofunction and DISC1 alteration are implemented in the neuropathology of schizophrenia (Callicott et al., 2005; Geddes et al., 2014), the mechanism of their possible interactions are not clear. In fact, dysfunction of GluN2B is involved in a number of severe mental disorders (Moghaddam and Javitt, 2012; Paoletti et al., 2013). GluN2B is found to be over-activated in Alzheimer's disease (Paoletti et al., 2013). Furthermore, GluN2B antagonists reduce amyloid β-induced synaptic deficits and impairs long term potentiation. (Rönicke et al., 2011) Conversely, GluN2B hypo-activity is found in hippocampus accompanied by cognitive impairment and memory loss in rats (Clayton et al., 2002). In clinical studies, GluN2B antagonists exacerbate the schizophrenia-like symptoms in both healthy people and patients (Moghaddam and Javitt, 2012), while antipsychotic drug olanzapine can activate GluN2B via phosphorylation of the GluN2B at Y1472 (Zhang et al., 2016).

Moreover, GluN2B has a number of phosphorylation sites, which could be responsible for GluN2B function. For example, phosphorylation of Y1472 attenuated the internalization of GluN2B (Roche et al., 2001). In this paper (Zhou et al., 2018), GluN2B changes were found, which could be due to alterations of the internalization. An early study has shown that Casein Kinase 2 (CK2) might be responsible for this internalization (Sanz-Clemente et al., 2010), therefore a detection of CK2 might help to clarify the internalization process of GluN2B.

In addition, due to the slow deactivation kinetics of GluN2B containing NMDARs (Cull-Candy and Leszkiewicz, 2004), activated synapses with GluN2B type NMDARs exhibit a strong Ca^2+^ influx. The Ca^2+^ influx in turn regulates downstream Ca^2+^-dependent proteins such as CaMKII and leads to activity-induced gene expression, synaptic plasticity and neurite growth (Sanz-Clemente et al., 2013; Barcomb et al., 2016). Thereafter, GluN2B-containing NMDARs regulate neurite growth and synaptic plasticity (Brigman et al., 2010).

It is noteworthy that GluN2B interacts with dopamine receptor D2 receptor (D2R) (Fan et al., 2014). D2R can physically interact with GluN2B in the post synaptic density (PSD) of the striatum in rats (Liu et al., 2006). Meanwhile, the binding of GluN2B with D2R can significantly affect the phosphorylation of S1303 of GluN2B, and thus affect CaMKII activity and synaptic plasticity. The author also found the binding sequence of D2R is TKRSSRAFRA (amino acid position 225–234) located in the third intracellular loop of D2R. Co-immunoprecipitation study also confirmed that GluN2B and D2R are tightly associated. Moreover, the activation of D2R by cocaine can significantly increase this complex formation and thus reduces NMDAR mediated currents due to dephosphorylated S1303 of the GluN2B subunit (Liu et al., 2006). Furthermore, another study also demonstrated D2R over-activation reduces spine density via GluN2B-dependent pathways in mice (Jia et al., 2013).

The recent manuscript of Zhou et al. showed that GluN2B hypofunction is highly related to the reduction of DISC1. Interestingly, DISC1 binds to D2R, and the binding site is located at amino acid positions 211–225 (Su et al., 2014), which is close to the GluN2B binding site and also located in the third intracellular loop of D2R. Primary cell culture studies have shown that D2R and DISC1 can form a complex, which is regulated by the activity of D2R (Su et al., 2014). Studies have shown that increased numbers of D2R-DISC1 complexes can be identified in post-mortem brains from schizophrenia patients and that interruption of D2R-DISC1 complex is correlated with antipsychotic effects in several mouse models (Dahoun et al., 2017). Additionally, phosphorylation of GSK-3α/β has been confirmed to be affected by D2R-DISC1 complex (Su et al., 2014). Therefore, GluN2B might interact with DISC1 through D2R and regulate its downstream GSK-3α/β signaling pathways. Furthermore, it is well known that D2R is one of the main targets of psychotherapy for schizophrenia and virtually all antipsychotic drugs have D2R antagonistic properties. Therefore, the involvement of D2R can connect DISC1 and GluN2B and provide a potential target for schizophrenia therapy.

## Author contributions

All authors listed have made a substantial, direct and intellectual contribution to the work, and approved it for publication.

### Conflict of interest statement

The authors declare that the research was conducted in the absence of any commercial or financial relationships that could be construed as a potential conflict of interest.

## References

[B1] Barcomb K. Hell J. W. Benke T. A. Bayer K. U. (2016). The CaMKII/GluN2B protein interaction maintains synaptic strength. J. Biol. Chem. 291, 16082–16089. 10.1074/jbc.M116.734822 27246855 PMC4965558

[B2] Brigman J. L. Wright T. Talani G. Prasad-Mulcare S. Jinde S. Seabold G. K.. (2010). Loss of GluN2B-containing NMDA receptors in CA1 hippocampus and cortex impairs long-term depression, reduces dendritic spine density, and disrupts learning. J. Neurosci. 30, 4590–4600. 10.1523/JNEUROSCI.0640-10.2010 20357110 PMC2869199

[B3] Burgdorf J. Zhang X. L. Nicholson K. L. Balster R. L. Leander J. D. Stanton P. K.. (2013). GLYX-13, a NMDA receptor glycine-site functional partial agonist, induces antidepressant-like effects without ketamine-like side effects. Neuropsychopharmacology 38, 729–742. 10.1038/npp.2012.246 23303054 PMC3671991

[B4] Callicott J. H. Straub R. E. Pezawas L. Egan M. F. Mattay V. S. Hariri A. R.. (2005). Variation in DISC1 affects hippocampal structure and function and increases risk for schizophrenia. Proc. Natl. Acad. Sci. U.S.A. 102, 8627–8632. 10.1073/pnas.0500515102 15939883 PMC1143583

[B5] Clayton D. A. Mesches M. H. Alvarez E. Bickford P. C. Browning M. D. (2002). A hippocampal NR2B deficit can mimic age-related changes in long-term potentiation and spatial learning in the Fischer 344 rat. J. Neurosci. 22, 3628–3637. 10.1523/JNEUROSCI.22-09-03628.2002 11978838 PMC6758397

[B6] Cull-Candy S. G. Leszkiewicz D. N. (2004). Role of distinct NMDA receptor subtypes at central synapses. Sci. STKE 2004:re16. 10.1126/stke.2552004re16 15494561

[B7] Dahoun T. Trossbach S. V. Brandon N. J. Korth C. Howes O. D. (2017). The impact of Disrupted-in-Schizophrenia 1 (DISC1) on the dopaminergic system: a systematic review. Transl. Psychiatry 7:e1015. 10.1038/tp.2016.282 28140405 PMC5299392

[B8] Fan X. Jin W. Y. Wang Y. T. (2014). The NMDA receptor complex: a multifunctional machine at the glutamatergic synapse. Front. Cell. Neurosci. 8:160. 10.3389/fncel.2014.00160 24959120 PMC4051310

[B9] Geddes A. E. Huang X. F. Newell K. A. (2014). GluN2B protein deficits in the left, but not the right, hippocampus in schizophrenia. BMC Psychiatry 14:274 10.1186/s12888-014-0274-z 25292222 PMC4195867

[B10] Jia J. M. Zhao J. Hu Z. Lindberg D. Li Z. (2013). Age-dependent regulation of synaptic connections by dopamine D2 receptors. Nat. Neurosci. 16, 1627–1636. 10.1038/nn.3542 24121738 PMC3832846

[B11] Liu X. Y. Chu X. P. Mao L. M. Wang M. Lan H. X. Li M. H.. (2006). Modulation of D2R-NR2B interactions in response to cocaine. Neuron 52, 897–909. 10.1016/j.neuron.2006.10.011 17145509

[B12] Moghaddam B. Javitt D. (2012). From revolution to evolution: the glutamate hypothesis of schizophrenia and its implication for treatment. Neuropsychopharmacology 37, 4–15. 10.1038/npp.2011.181 21956446 PMC3238069

[B13] Moskal J. R. Burch R. Burgdorf J. S. Kroes R. A. Stanton P. K. Disterhoft J. F.. (2014). GLYX-13, an NMDA receptor glycine site functional partial agonist enhances cognition and produces antidepressant effects without the psychotomimetic side effects of NMDA receptor antagonists. Expert Opin. Investig. Drugs 23, 243–254. 10.1517/13543784.2014.852536 24251380 PMC4721220

[B14] Moskal J. R. Kuo A. G. Weiss C. Wood P. L. O'Connor Hanson A. Kelso S.. (2005). GLYX-13: a monoclonal antibody-derived peptide that acts as an N-methyl-D-aspartate receptor modulator. Neuropharmacology 49, 1077–1087. 10.1016/j.neuropharm.2005.06.006 16051282

[B15] Paoletti P. Bellone C. Zhou Q. (2013). NMDA receptor subunit diversity: impact on receptor properties, synaptic plasticity and disease. Nat. Rev. Neurosci. 14, 383–400. 10.1038/nrn3504 23686171

[B16] Parsons C. G. Danysz W. Quack G. (1999). Memantine is a clinically well tolerated N-methyl-D-aspartate (NMDA) receptor antagonist–a review of preclinical data. Neuropharmacology 38, 735–767. 10.1016/S0028-3908(99)00019-2 10465680

[B17] Roche K. W. Standley S. McCallum J. Dune Ly C. Ehlers M. D. Wenthold R. J. (2001). Molecular determinants of NMDA receptor internalization. Nat. Neurosci. 4, 794–802. 10.1038/90498 11477425

[B18] Rönicke R. Mikhaylova M. Ronicke S. Meinhardt J. Schröder U. H. Fandrich M.. (2011). Early neuronal dysfunction by amyloid beta oligomers depends on activation of NR2B-containing NMDA receptors. Neurobiol. Aging 32, 2219–2228. 10.1016/j.neurobiolaging.2010.01.011 20133015

[B19] Sanz-Clemente A. Gray J. A. Ogilvie K. A. Nicoll R. A. Roche K. W. (2013). Activated CaMKII couples GluN2B and casein kinase 2 to control synaptic NMDA receptors. Cell Rep. 3, 607–614. 10.1016/j.celrep.2013.02.011 23478024 PMC3615108

[B20] Sanz-Clemente A. Matta J. A. Isaac J. T. Roche K. W. (2010). Casein kinase 2 regulates the NR2 subunit composition of synaptic NMDA receptors. Neuron 67, 984–996. 10.1016/j.neuron.2010.08.011 20869595 PMC2947143

[B21] Stanton P. K. Potter P. E. Aguilar J. Decandia M. Moskal J. R. (2009). Neuroprotection by a novel NMDAR functional glycine site partial agonist, GLYX-13. Neuroreport 20, 1193–1197. 10.1097/WNR.0b013e32832f5130 19623090

[B22] Su P. Li S. Chen S. Lipina T. V. Wang M. Lai T. K.. (2014). A dopamine D2 receptor-DISC1 protein complex may contribute to antipsychotic-like effects. Neuron 84, 1302–1316. 10.1016/j.neuron.2014.11.007 25433637

[B23] Zhang Q. Yu Y. Huang X. F. (2016). Olanzapine prevents the PCP-induced reduction in the neurite outgrowth of prefrontal cortical neurons via NRG1. Sci. Rep. 6:19581. 10.1038/srep1958 26781398 PMC4726088

[B24] Zhang X. L. Sullivan J. A. Moskal J. R. Stanton P. K. (2008). A NMDA receptor glycine site partial agonist, GLYX-13, simultaneously enhances LTP and reduces LTD at Schaffer collateral-CA1 synapses in hippocampus. Neuropharmacology 55, 1238–1250. 10.1016/j.neuropharm.2008.08.018 18796308 PMC2661239

[B25] Zhou D. Lv D. Wang Z. Zhang Y. Chen Z. Wang C. (2018). GLYX-13 ameliorates schizophrenia-like phenotype induced by MK-801 in mice: role of hippocampal NR2B and DISC1. Front. Mol. Neurosci. 11:121. 10.3389/fnmol.2018.00121 29695955 PMC5904356

